# P-818. Third-Generation Cephalosporin Resistant Enterobacterales Without Genetic Detection on the BIOFIRE Blood Culture Identification 2 Panel: A De-escalation Tool

**DOI:** 10.1093/ofid/ofaf695.1026

**Published:** 2026-01-11

**Authors:** Nadeem Baalbaki, Kaitlyn Bowden, Debra Chew

**Affiliations:** University Hospital, Newark, NJ; University Hospital, Newark, NJ; Rutgers New Jersey Medical School, Newark, New Jersey

## Abstract

**Background:**

The BIOFIRE Blood Culture Identification 2 Panel (BCID2) is a sensitive ( >99%) in vitro diagnostic tool that rapidly identifies select organisms and resistance genes (RGs) via nucleic acid amplification. It detects the predominant ESBL gene, CTX-M, along with other types of RGs. However, less common resistance mechanisms, not detected by the BCID2, may confer ceftriaxone-resistant (CRO-R) and cefepime (FEP) susceptible isolates. Ceftriaxone and FEP are commonly used for empiric treatment of Enterobacterales with similar susceptibility profiles exhibited by select species institutionally and globally. Determining the incidence of CRO-R Enterobacterales blood isolates without RGs detection via BCID2 may support early de-escalation.

**Methods:**

This was a retrospective review of all blood isolates in adult patients cultured at our institution from January 2023 to December 2024 with growth of either *E. coli*, *K. pneumoniae*, *K. oxytoca*, or *P. mirabilis*. Isolates were included if they were CRO-R and the organism was detected on the BCID2 panel. Isolates were excluded if the BCID2 did not detect the organism, if the BCID2 was not performed, if the same organism was cultured within 48 hours of a previous culture or if there were polymicrobial findings with > 1 ESBL organism. We sought to describe the incidence of CRO-R Enterobacterales with RGs negative BCID2 results.

**Results:**

A total of 432 blood isolates were obtained. Of these, 95 (22%) were identified as CRO-R and reviewed in our study. The overall incidence of any CRO-R isolate without RGs detected via BCID2 was 1.6%. Five isolates (1.2%) were flagged as ESBL without RGs detected. Two isolates (0.5%) were CRO-R without an ESBL flag via MicroScan and without any RGs detected; these 2 isolates were both E. coli (Table 1), and both remained FEP susceptible.

**Conclusion:**

These findings suggest that early de-escalation from FEP to CRO using the BCID2 panel in the setting of RG-negative results with *E. coli*, *K. pneumoniae*, *K. oxytoca*, or *P. mirabilis* may be clinically appropriate, with only a 0.5% risk of inappropriate de-escalation at our institution. Further studies are needed to confirm this due to varying institution-specific susceptibility patterns.

**Disclosures:**

All Authors: No reported disclosures

